# Case report: *Streptococcus pneumoniae* pneumonia characterized by diffuse centrilobular nodules in both lungs

**DOI:** 10.3389/fmed.2022.1007160

**Published:** 2023-01-10

**Authors:** Fangqi Zhang, Shaowen Qin, Fei Xia, Congzheng Mao, Long Li

**Affiliations:** Department of Pulmonary and Critical Care Medicine, General Hospital of Central Theater Command of the People’s Liberation Army, Wuhan, Hubei, China

**Keywords:** *Streptococcus pneumoniae*, pneumonia, chest computed tomography, centrilobular nodules, case report

## Abstract

**Background:**

*Streptococcus pneumoniae* (*S. pneumoniae*) is the most common pathogen in community-acquired pneumonia (CAP) and takes the form of lobar pneumonia as typical computed tomography (CT) findings. Various patterns of radiological manifestation have also been reported in patients with *S. pneumoniae* pneumonia; however, the appearance of diffuse centrilobular nodules in both lungs is rarely reported.

**Case presentation:**

We report the case of a patient with a history of chronic lymphocytic leukemia (CLL) for 9 years who presented with new-onset fever, cough, excess sputum, and shortness of breath for 1 week. He was given intravenous antibacterial (cephalosporin) treatment for 4 days, but his condition did not improve and dyspnea became more serious. The chest CT indicated diffuse centrilobular nodules in both lungs at admission. Patient’s bronchoalveolar (BAL) fluid was sent for metagenomic next-generation sequencing, which only supported a diagnosis of *S*. *pneumoniae* infection. His condition improved gradually after antimicrobial treatment (moxifloxacin) and a follow-up CT showed that the diffuse centrilobular nodules in both lungs were absorbed completely.

**Conclusion:**

This case highlights a rare CT presentation of *S. pneumoniae* pneumonia that should alert clinicians, so as to avoid taking unnecessary treatment measures.

## Introduction

*Streptococcus pneumoniae* (*S. pneumoniae*) remains one of the most common causes of bacterial community-acquired pneumonia (CAP), encompassing infections mild enough to be treated on an outpatient basis, as well as those requiring hospital care, or even intensive care unit admission (1). Pneumolysin is the major protein virulence factor of the *S. pneumoniae* and possesses both cytotoxic and proinflammatory properties (2). The toxin is located in the cytoplasm of the *S. pneumoniae*, as well as on the cell wall, and is released extracellularly following the autolysis of the pathogen during the later stages of growth, which resulted in the development of pneumonia restricted to the lobe (2, 3). Therefore, *S. pneumoniae* pneumonia takes the form of lobar pneumonia as typical computed tomography (CT) findings (4). Various patterns and distributions of radiological manifestation have also been reported in patients with *S. pneumoniae* pneumonia owing to the widespread use of antibiotics (5). Bronchopneumonia and associated centrilobular nodules were also not uncommon in CT findings of *S. pneumoniae* pneumonia cases (6, 7). However, these nodules were usually at the periphery of consolidation, or the lesions were localized to lung segment or lobe.

Herein, we report a rare case of *S. pneumoniae* pneumonia characterized by diffuse centrilobular nodules in both lungs, which adds to the body of knowledge about *S. pneumoniae*.

## Case presentation

**FIGURE 1:**
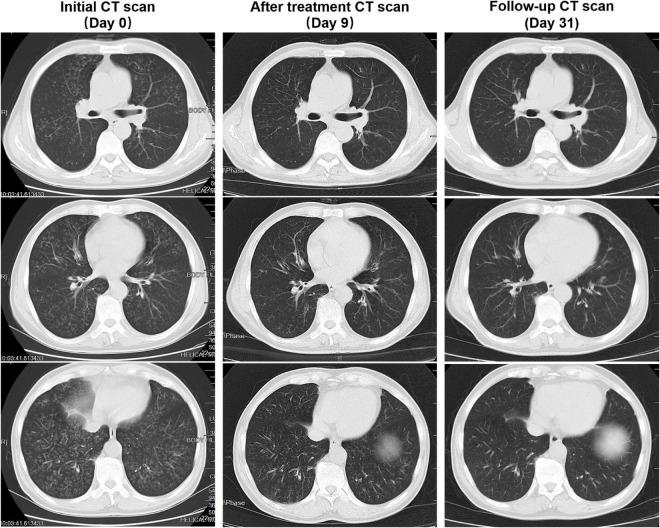
Patient’s computed tomography (CT) scan images at three time points. Initial CT scan (Day 0) showed that bilateral diffuse nodules separated by the fissures and pleura. Some of the nodules have a “tree-in-bud” appearance. After treatment, CT scan (Day 9) showed visible absorption of diffuse nodules in both lungs. Follow-up CT scan (Day 31) showed that the bilateral diffuse nodules absorbed completely.

**FIGURE 2:**
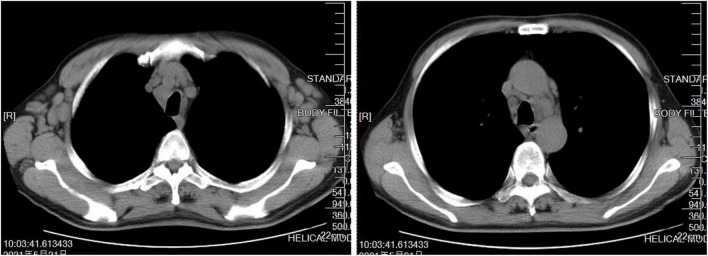
Mediastinal window of patient’s initial computed tomography (CT) scan showed multiple enlarged lymph nodes in mediastinum and bilateral axilla.

A 66-year-old man presented with fever, cough, excess sputum, and shortness of breath for 1 week. He was given intravenous antibacterial (cephalosporin) treatment for 4 days, but his condition did not improve and dyspnea became more serious. Therefore, the patient came to our hospital where chest CT showed diffuse centrilobular nodules in both lungs, some with a “tree-in-bud” appearance (Figure 1), and multiple enlarged lymph nodes in mediastinum and bilateral axilla (Figure 2). He was then admitted to the hospital.

The patient had a history of CLL for 9 years and had received several chemotherapies in the past. His condition was stable in the past year. He has a history of smoking for 30 years. Other medical history was denied. He had no bird exposure and no history of travel outside Wuhan, Hubei, where he lived.

At admission, physical examination revealed that temperature was 37.8°C, peripheral blood oxygen saturation (SpO2) was 87% on room air, respiratory rate was 31 breaths/min, and wet rales could be heard on auscultation of both lungs. Multiple soy-sized enlarged lymph nodes could be palpable on both sides of the neck and armpits. Laboratory tests revealed that arterial blood gases at 29% fraction of inspiration O2 (FiO2) showed partial pressure of oxygen (PaO2) 65.6 mmHg, partial arterial pressure of carbon dioxide (PaCO2) 22.8 mmHg, pH 7.43, and oxygenation index (OI; PaO2/FiO2) 226 mmHg. Blood cell analyses, C-reactive protein (CRP), and erythrocyte sedimentation rate (ESR) were examined (Table 1). T-cell spots of tuberculosis infection (T-SPOT.TB) were positive. The detection of mycobacterium tuberculosis in bronchoalveolar (BAL) fluid by polymerase chain reaction (PCR) was negative. Sputum and BAL fluid acid-fast staining were negative. Procalcitonin, immunoglobulin (Ig) E, *Mycoplasma pneumoniae* IgM, *Chlamydia pneumoniae* IgM, and 1, 3-beta-D glucan/galactomannan tests were normal. No pathogenic bacteria or fungi were detected in blood, sputum, and BAF fluid cultures. Lymphocyte typing were noted as follows: total T cell 6% (normal, 50–87%), total T-cell number 803/μl (normal, 955–2,860/μl), CD4 + Th-cell proportion 1% (normal, 21–51%), CD4 + Th-cell number 151/μl (normal, 550–1,440/μl), total B-cell 89% (normal, 3–19%), and total B-cell number 12,603/μl (normal, 90–560/μl).

**TABLE 1:** Laboratory parameters of patients at admission and before discharge.

Laboratory parameters	At admission	Before discharge
SpO2 (on room air)	87%	96%
WBC	17.1 × 10^9^/L	12.1 × 10^9^/L
EOS (EOS%)	0.10 × 10^9^/L (0.6%)	0.12 × 10^9^/L (1.0%)
NEU (NEU%)	4.98 × 10^9^/L (29.0%)	2.02 × 10^9^/L (16.7%)
LYM (LYM%)	11.59 × 10^9^/L (67.7%)	9.63 × 10^9^/L (79.7%)
CRP	89.69 mg/L	<0.449 mg/L
ESR	55 mm/h	1 mm/h

SpO2, peripheral blood oxygen saturation; WBC, white blood cell; EOS, eosinophil; NEU, neutrophil; LYM, lymphocyte; CRP, C-reactive protein; ESR, erythrocyte sedimentation rate.

The patient was diagnosed with severe pneumonia and type 1 respiratory failure. BAL fluid was collected and sent to the Shenzhen BGI Medical Test Laboratory for metagenomic next-generation sequencing (mNGS), which only supported a diagnosis of *S. pneumoniae* infection.

After admission, the patient’s condition improved gradually by giving antimicrobial treatment (moxifloxacin injection, 0.4 g, qd) and other comprehensive treatment measures, including airway clearance and oxygen support. Before discharge, SpO2, blood cell analyses, and related inflammatory markers were reexamined (Table 1), in which SpO2, CRP, and ESR were normal finally, and reexamination of the chest CT showed visible absorption of diffuse centrilobular nodules in both lungs (Figure 1). Three weeks after discharge, a follow-up CT showed that the diffuse centrilobular nodules in both lungs absorbed completely (Figure 1).

## Discussion

The differential diagnosis of diffuse centrilobular nodules is extensive but small airway diseases are by far the most likely cause, including infectious bronchiolitis, aspiration, hypersensitivity pneumonitis, respiratory bronchiolitis (RB), and follicular bronchiolitis (8). “Tree-in-bud” indicates the presence of dilated centrilobular bronchioles with lumen impacted by mucus, fluid, or pus and is associated with peribronchiolar inflammation (9). Centrilobular nodules showing “tree-in-bud” appearance are associated with airway infection in majority of patients, and the common pathogens include mycobacterium tuberculosis, non-tubercular mycobacteria (typically MAC), *Haemophilus influenzae*, *M. pneumoniae*, *Chlamydia*, and viral and airway invasive aspergillus. In this case, however, the patient’s BAL fluid was detected by mNGS, which only supported a diagnosis of *S. pneumoniae* infection. Although pathogens mNGS can detect bacteria, viruses, fungi, and parasites without bias, there may be some omissions in RNA virus due to sample storage and transportation problems. The possibility of co-infection with virus cannot be ruled out. However, in the absence of antiviral treatment, the rapid improvement in symptoms and CT imaging in patients with an impaired immune system suggests that the possibility of virus infection is unlikely.

*Streptococcus pneumoniae* pneumonia typically presents the form of homogeneous airspace consolidation, whereby alveolar lumens are filled with exudates containing leukocytes and alveolar walls are thickened by capillary congestion and edema (4, 5). Associated centrilobular nodules were not uncommon. Previous reports have found that 27–48% of patients with *S. pneumoniae* pneumonia exhibited centrilobular nodules on CT scans (6, 7); however, centrilobular nodules were usually at the periphery of consolidation or the lesions were localized to lung segment or lobe. To the best of our knowledge, no cases presenting diffuse centrilobular nodules in both lungs of patients with *S. pneumoniae* pneumonia have been published.

The patient had a history of CLL for 9 years and smoking for 30 years. CLL is characterized by the clonal proliferation and accumulation of mature and typically CD5 + B-cells within the blood, bone marrow, lymph nodes, and spleen (10). With an impaired immune system, patients with CLL often develop infectious complication, in which *S. pneumoniae* pneumonia is not uncommon. Unfortunately, the information of imaging patterns available about *S. pneumoniae* infection in a patient with CLL is limited. Cigarette smoking is also a common risk factor for *S. pneumoniae* pneumonia (1). The mechanisms of this association possibly include altered ciliary motility, increased nasopharyngeal carriage of organisms, altered alveolar macrophage function, and increased epithelial permeability (11). Meanwhile, there is strong evidence supporting a causal role of cigarette smoking in the development of RB and RB-associated interstitial lung disease (RB-ILD), which are also characterized by diffuse centrilobular nodules (12, 13). However, it is unknown whether these comorbid conditions contribute to this rare imaging appearance in a patient with *S. pneumoniae* infection. We hypothesize that this may have been implicated in this patient, and further studies are warranted.

This case highlights a rare CT presentation of *S. pneumoniae* pneumonia that should alert clinicians, so as to avoid taking unnecessary treatment measures.

## Data availability statement

The original contributions presented in this study are included in the article/supplementary material, further inquiries can be directed to the corresponding author.

## Ethics statement

Written informed consent was obtained from the individual(s) for the publication of any potentially identifiable images or data included in this article.

## Author contributions

FZ and LL generated the concept. FZ and SQ drafted the manuscript. FZ was the consultant in charge of the patient. FX, CM, and LL revised the original draft critically for important intellectual content. All authors contributed to the article and approved the submitted version.
